# Sustaining patient and public involvement and engagement in research

**DOI:** 10.1111/hex.12848

**Published:** 2018-11-22

**Authors:** Carolyn A. Chew‐Graham

**Affiliations:** ^1^ Research Institute Primary Care and Health Sciences Faculty of Medicine and Health Sciences Keele University Keele Staffordshire UK

The involvement of patients and the wider public in health research has been reported increasingly over the last decades. The rationale for including a patient and public perspective in research, including in systematic reviews and qualitative studies, has been advocated by authorities such as the National Institute for Health Research (NIHR) advisory group, INVOLVE.^1^

*Health Expectations* prioritizes the publication of manuscripts where patient and public involvement and engagement (PPIE) has contributed to the design and conduct of the research, provided input to interpretation of findings and advised on dissemination activities.

In this edition, Hannigan's viewpoint article offers a perspective on the role of PPIE on studies using quantitative methods, highlighting the training needs for both patients and the public, but also statisticians, but emphasizing how PPIE can increase impact of this type of research.

Hawke et al provide practical recommendations to help researchers engage young people in meaningful ways in academic research, from initial planning to project completion, empathizing the value of early engagement.

Collins et al describe the utility of the public Involvement Impact Assessment Framework (PiiAF), previously developed by the authors, as a resource to support research teams in assessing the impact of public Involvement in a mental health research centre. The authors emphasize that PPIE had been integral to the development of the PiiAF and emphasize the importance of public involvement in the Spectrum Centre (the research centre under discussion). The team note that adequate funding, costed into funding applications, is required to support PPIE activity.

Having a dedicated PPIE team is key to support research which is relevant to the needs of patients and the public. Two primary care institutions in the United Kingdom have shared their views on what is needed to support and sustain PPIE in their research.

Rebecca Morris (Research Fellow ‐ Patient and Public Involvement and Engagement, Keele University), Angela Ruddock and Moira Lyons (Chair and co‐chair of PRIMER, University of Manchester) describe the PRIMER^2^ (Primary Care Research in Manchester Engagement Resource) group, a PPIE group which has worked with researchers at the Centre for Primary Care at the University of Manchester since 2008. The members are an active part of the department, suggesting topics for research, helping to shape research ideas, contributing to funding applications, and then working with researchers as co‐investigators to develop, deliver and disseminate research projects. PRIMER members and researchers work together to advise on best practice in PPIE, hold workshops to advance critical thinking and discussion about public involvement, and work with national and international organizations to promote, discuss and involve PPIE in research. Members have also co‐developed and co‐delivered, with researchers, a series of training modules from introductory courses to masterclasses and support each other, undergraduate, postgraduate and health services researchers to establish an approach to PPIE which has been internationally recognized.

Steven Blackburn (Research Fellow ‐ Patient and Public Involvement and Engagement, Keele University) describes the Research User Group (RUG)^3^ at Keele University as integral to the Research Institute for Primary Care and Health Sciences, ensuring that its research activities are shaped and co‐produced by patients. Study teams involve RUG members throughout the research cycle and in a range of study design, including clinical trials, qualitative studies, systematic reviews and epidemiological (survey and medical record reviews) studies. PPIE activity is part of the research and implementation culture and depends on dedicated RUG members, engaged staff, senior support, and an established PPIE infrastructure.^4^ RUG members may join study advisory groups, contributing to specific elements of a study, such as agreeing priorities for research, recruitment strategies, outcome measures, participant information, data analysis and interpretation and dissemination. In addition, RUG members may be supported to be funding application co‐applicants, and members of Study Steering Committees. The Keele RUG has been selected to be a “test‐bed site” for testing standards on public engagement.^5^

Blackburn et al^6^ describe a Costs and Consequences Framework which enables an assessment of the benefits, harms and costs (financial and non‐financial) of PPIE.

In this edition of *Health Expectations*, Larsen and Saagvarg describe an action research project, with patient co‐researchers supporting data collection and analysis. This study illustrates the integration of the public voice in research and is exactly the sort of manuscript welcomed by HEX.
